# *Cropped, Drosophila* transcription factor AP-4, controls tracheal terminal branching and cell growth

**DOI:** 10.1186/s12861-015-0069-6

**Published:** 2015-04-15

**Authors:** Matthew Man-Kin Wong, Ming-Fai Liu, Sung Kay Chiu

**Affiliations:** Department of Biomedical Sciences, City University of Hong Kong, Tat Chee Avenue, Kowloon Tong, Hong Kong; Department of Biology and Chemistry, City University of Hong Kong, Tat Chee Avenue, Kowloon Tong, Hong Kong; Department of Biochemistry and Howard Hughes Medical Institute, Stanford University School of Medicine, Stanford, CA 94305-5307 USA

**Keywords:** *Drosophila*, Transcription factor AP-4, Cellular branching, Cell growth, Trachea, Myc

## Abstract

**Background:**

Endothelial or epithelial cellular branching is vital in development and cancer progression; however, the molecular mechanisms of these processes are not clear. In *Drosophila*, terminal cell at the end of some tracheal tube ramifies numerous fine branches on the internal organs to supply oxygen. To discover more genes involved in terminal branching, we searched for mutants with very few terminal branches using the Kiss enhancer-trap line collection.

**Results:**

In this analysis, we identified *cropped (crp)*, encoding the *Drosophila* homolog of the transcription activator protein AP-4. Overexpressing the wild-type *crp* gene or a mutant that lacks the DNA-binding region in either the tracheal tissues or terminal cells led to a loss-of-function phenotype, implying that *crp* can affect terminal branching. Unexpectedly, the ectopic expression of *cropped* also led to enlarged organs, and cell-counting experiments on the salivary glands suggest that elevated levels of AP-4 increase cell size and organ size. Like its mammalian counterpart, *cropped* is controlled by dMyc, as ectopic expression of dMyc in terminal cells increased cellular branching and the Cropped protein levels in vivo.

**Conclusions:**

We find that the branching morphogenesis of terminal cells of the tracheal tubes in *Drosophila* requires the dMyc-dependent activation of Cropped/AP-4 protein to increase the cell growth of terminal cells.

**Electronic supplementary material:**

The online version of this article (doi:10.1186/s12861-015-0069-6) contains supplementary material, which is available to authorized users.

## Background

In *Drosophila*, atmospheric oxygen enters the body via the spiracles and then is transported to each segment of the body through an extensive tubular tracheal network. These tubules are initially formed by an invagination of the epithelial cells. This epithelial cell migration is initiated and directed by Branchless, the *Drosophila* homolog of the mammalian Fibroblast Growth Factor (FGF) [1], which acts as a chemoattractant or motogen, and is secreted from the cell clusters surrounding the tracheal placoid in each segment of the body. The tubes cease further extension when the cells at the tips of the tube meet the FGF-secreting cells [2,3].

The cells with the highest FGF activity take up the leading position at the end of a tracheal branch, whereas the other cells with less FGF activity form the stalk of the branches [4]. The tracheal lumens from the primary and secondary branches extend into the cell bodies of terminal cells. At stage 16, near the end of embryogenesis, a single lumen containing branch is formed by the extension of a long cytoplasmic projection from terminal cell along the surface of somatic muscles. During larval development, this single terminal branch ramifies extensively into many additional fine branches that later develop lumens. Recent studies show that the PAR-polarity complex, including Par-6, Par-3, Cdc42, and aPKC, is involved in the subcellular branching of terminal cells [5].

Most of these tracheal branches supply oxygen to identical sets of targets, but certain branches, such as the visceral branches, tracheate to unique organs and do not develop a repetitive pattern of branching. The density of terminal branches serving a target tissue depends on the oxygen requirements of the tissue [6]. A detailed examination of all terminal branches revealed that most of the cells in the body are either directly in contact with or very close to a terminal branch [3]. Jarecki et al. (1999) demonstrated that hypoxia induces the formation of additional terminal branches through an increase in the Branchless FGF levels in the tissues, which correlate well with the density of branches [6]. Moreover, the over-expression of Branchless FGF in the target tissues increases the number of terminal branches, as does hypoxia. Centanin and colleagues demonstrated that the hypoxia-induced generation of excess terminal branches is mediated by the accumulation of the Hypoxia-Inducible Factor (HIF)-α homolog Sima in terminal cells, leading to the induction of *breathless* [7]. In addition, *Drosophila* Serum Responsive Factor (DSRF) or Blistered is involved in terminal branching, which is induced by FGF [1,8]. DSRF is necessary for the progression of terminal branching after the initial elongation of the cell and lumen [9]. Many identified genes related to terminal branching were found in genetic screens with their tracheal expression pattern during embryogenesis. More recently, several studies on the direct identification of genes involved in larval tracheal branching have started to reveal more about the genetic control cascades on the branching mechanism [10,11].

To gain further insight into the regulation of the formation of tracheal terminal branches during larval stages, we carried out a genetic screen on the Kiss collection of P-element enhancer trap mutants with tracheal terminal branching defects, taking advantage of the P-element insertion into genes, which can be identified and cloned relatively easily [12,13]. In the screen, we discovered several mutants that have severe truncation of terminal branches, and in this paper, we studied one of these mutants, called *cropped/crp*, which encodes the *Drosophila* homolog of the mammalian transcription factor AP-4. We showed that *crp* acts mainly in terminal cells, and that insertion and point mutations in *crp* lead to truncation in terminal branches. Besides controlling tracheal cellular branching, overexpressing *crp* leads to increase in cell size and disruption in *crp* function results in developmental defect and cell death in the eyes and salivary glands. In addition, we show that dMyc may be the upstream regulator of *crp* in the induction of terminal branching. This study demonstrates that Crp, as a downstream regulator of dMyc, is a pleiotropic transcription factor that controls cellular branching, cell growth and apoptosis in various organs during *Drosophila* larval development.

## Results

### Identification and genetic characterization of *cropped (crp)*

**Figure 1:**
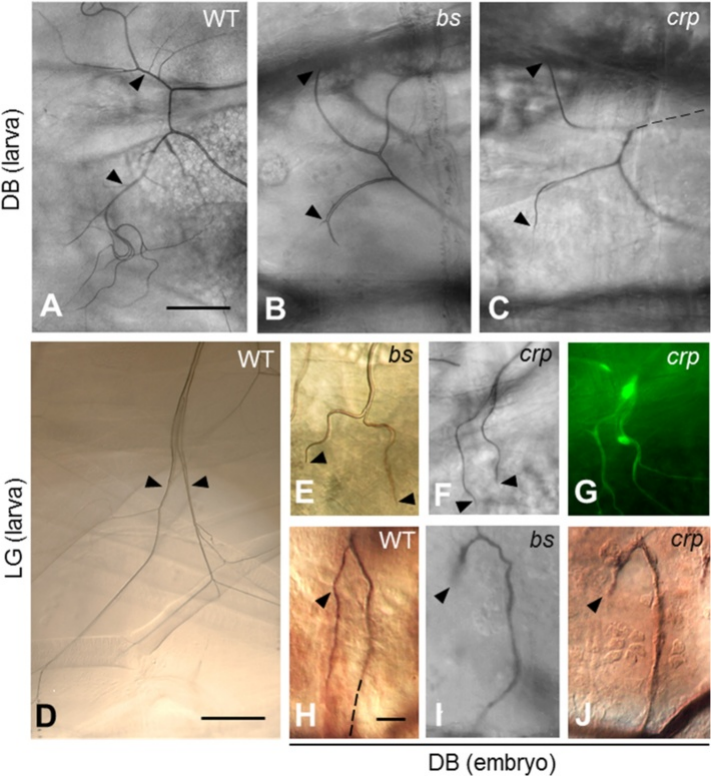
The tracheal phenotypes of the *cropped* mutant compared with the *blistered/pruned* mutant. **(A)** A pair of dorsal branches (DB, arrowheads) in a wild-type third-instar larva. The air-filled terminal branches ramify extensively on the dorsal muscles. **(B)** The same view of a *blistered* ^*ex84*^ homozygote. Note the absence of terminal branches beyond the arrowheads where the nuclei of terminal cells are. **(C)** A *crp* ^*k10415*^ homozygote. Note the absence of terminal branches beyond the arrowheads, as in the *blistered* mutant. **(D)** A pair of lateral trunk LG branches (arrowheads) in a wild-type third-instar larva. **(E)** The same view of a *blistered* ^*ex84*^ homozygote. Note the absence of terminal branches beyond the arrowheads. **(F)** A *crp* ^*k10415*^; *btl-Gal4/UAS-GFP* larva. **(G)** A fluorescence image of **(F)** showing the GFP-labeled tracheal cell cytoplasmic extensions. **(H)** A dorsal branch of a stage 16/17 wild-type embryo stained with mAb 2A12 to show the tracheal lumen. The arrowhead indicates the position of the terminal nucleus. The dashed line shows the continuation of the base of the dorsal branch out of the plane of focus. **(I)** The same view of a *blistered* ^*ex84*^ mutant. **(J)** A *crp* ^*k10415*^ mutant. The scale bars for **A**-**D** = 30 μm, for **E**-**J** = 10 μm.

In a genetic screen for mutations that affect the outgrowth of tracheal terminal branches, we identified three independent *P[lacZ]* enhancer trap mutations that caused a substantial reduction in the number of terminal branches in larvae. One of these mutants, (*l(2)k10415*), which we call *cropped* (*crp*)*,* is described in this paper. In wild-type animals, the first terminal branches sprout at the end of embryogenesis, and, these branches ramify into extensive networks of fine branches throughout larval life (Figure 1A). In homozygous *crp*^*k10415*^ mutants, the early stages of tracheal development were normal, but most of the terminal branches seldom extend beyond the cell body (Figure 1C), similar to what is observed in *blistered/pruned* mutants (Figure 1B). The counting of dorsal branches included only from segments 3 to 9 because they were clearly visible under the microscope. In 54% of the dorsal branches (total DBs counted; n = 229), only one terminal branch remained, and the ones that did develop (14%, DBs; n = 229) were much shorter than normal; the remainder of the DBs exhibited a less severe and variable reduction in branching. The visceral, ganglionic, and lateral trunk terminal branches were affected similarly; 67% of the lateral G (LG) branch split into two branches but with few finer terminal branches (Figure 1F and G). A tracheal cytoplasmic GFP marker showed that terminal cells in the *crp*^*k10415*^ mutant extended fewer (N = 2.3 ± 0.68) cytoplasmic processes than normal (N = 15.7 ± 2.7) (Figure 1G) and that many of the processes that formed did not develop an air-filled lumen (Figure 1F vs. Figure 1D). The larval terminal branching defect of *crp*^*k10415*^ resembles that of *blistered* mutants phenotypically (Figure 1B and E), and like *blistered* mutants, the *crp*^*k10415*^ mutant also affected the outgrowth of the first terminal branches of DB that form at the end of embryogenesis (Figure 1I and J), suggesting that *cropped* is necessary for the embryonic and larval tracheal terminal branching. Tracheal terminal branches appear to be the only tissue affected, as the other tissues, including the muscles, gut, central nervous system, salivary glands, and imaginal discs, were grossly intact in the mutants.

The *P[lacZ]* insertion in *crp*^*l(2)k10415*^ maps to the cytological position 35 F1-2 and forms part of the *l(2)35Fd* complementation group, which includes ~30 other transposon alleles and 2 EMS-induced alleles [14,15]. We analyzed the tracheal phenotypes of seven transposon alleles and the one EMS allele that was available at that time, *35Fd2/RAR46*. All of the homozygotes showed similar embryonic and larval tracheal phenotypes to *crp*^*l(2)k10415*^, although the *l(2)k07829* phenotype was much weaker. *35Fd2/RAR46* did not survive to late larval stages (data not shown). Based on these observations, *crp*^*k00809*^ appeared to be a null allele for the tracheal function because *crp*^*k00809*^ homozygotes (87.5% of DBs severely affected, n = 56) displayed as severe a phenotype as *crp*^*k00809*^ hemizygotes (*k00809/Df(2 L)r10*; 81%, n = 121), as did *k10415/k00809* trans-heterozygotes (83%, n = 59) and *k10415* hemizygotes (*crp*^*k10415*^*/Df(2 L)r10*; 85%, n = 106). There was no tracheal phenotype in *crp*^*k10415*^*/+* heterozygotes.

### *Cropped* encodes the *Drosophila* homolog of the mammalian transcription factor AP-4

**Figure 2:**
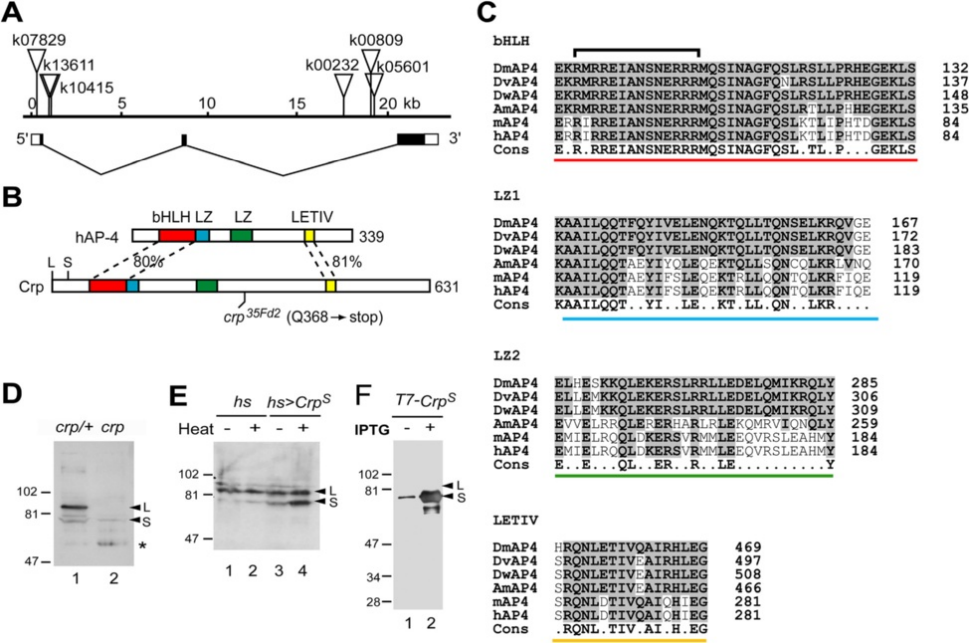
The structure of the *cropped* locus and its gene products. **(A)** The structure of the *crp* locus. The positions of *P*[*lacZ, w*] insertions (inverted triangles) in *crp* mutants are shown. The boxes at the bottom show the exons and the black boxes indicate the coding region. **(B)** Schematics of the primary structure of the Cropped and human AP-4 (hAP-4) proteins. The bHLH DNA-binding and dimerization domain (red), two leucine zippers (LZ, blue and green), and a region containing a conserved motif LETIV (yellow) are shown. The percent sequence identity between Cropped and hAP-4 is shown for the regions indicated. The positions of the in-frame initiator codons corresponding to the long (L) and short (S) Cropped protein isoforms are shown. The mutation of *crp* ^*35Fd2*^ is indicated. **(C)** An alignment of the primary sequences of the 4 conserved domains of Cropped from *D. melanogaster*, *D. virilis*, *D. willistoni*, *Apis mellifera*, mouse AP-4, and hAP-4 is shown. The consensus sequence (Cons) is shown on the bottom. The bracket indicates the basic DNA binding region, which is deleted in DN-Cropped. **(D)** Immunoblots of equivalent amounts of protein extracts from *crp* ^+^ (*crp* ^*35Fd2/+*^) and *crp* ^*−*^ (*crp* ^*35Fd2*^/*crp* ^*35Fd2*^) first instar larvae using anti-Cropped antiserum. The 85-kD band corresponds to the long Cropped isoform (L), and the 78-kD band corresponds to the short isoform (S). The lower molecular band (asterisk) is variable and may also be a cross-reacting species. **(E)** Immunoblots, as above, of protein extracts from control (*hs-Gal4* alone) and *UAS-cropped* ^*Short*^; *hs-Gal4* adult flies subjected to heat-shock at 35°C for 1 hr to induce transgene expression. **(F)** An immunoblot of extracts of *E. coli* transformed with an inducible T7-*cropped(short)* vector before (−) and after (+) induction with IPTG. The Cropped (short) protein migrates at the same position as the short isoform in *Drosophila* cell extract.

The genomic DNA flanking the insertion sites of the *crp*^*k10415*^ allele and five other *P[lacZ]* alleles was isolated by plasmid rescue. DNA sequencing showed that the transposon insertion sites were distributed over 20 kbp in the 35 F region of the *Drosophila* genome (Figure 2A). The high susceptibility of the two introns to transposon insertion suggests that this genomic region is likely accessible chromatin [16]. Four lines of evidence demonstrate that this transcription unit corresponds to *crp*. First, all six of the characterized *P[lacZ]* alleles described above are insertions in this transcription unit, and all of these alleles show patterns of lacZ expression in the embryo and larva that are similar to the expression pattern of this transcript (Additional file 1: Table S1). Second, genomic DNA sequencing of the *crp* EMS allele (*35Fd2/RAR46*) and two sibling control strains, *l(2)35Dh[AS64]* and *l(2)35Di[RAR8]*, identified a nonsense mutation (Q to stop-codon) in the *35Fd2* allele, which truncates the predicted protein (Figure 2B). Third, the protein products of this transcription unit were absent in *crp*^*35Fd2*^ homozygotes (Figure 2D). Finally, the tracheal phenotype could be mimicked by the expression of a dominant-negative form of this protein in the tracheal tissue (see below).

The *crp* cDNA encodes a 631-amino acid (calculated MW of 69 kDa) protein with high sequence homology to the human Activator Protein 4 (AP-4, Figure 2B). Using the protein sequence of Cropped to blast against protein sequences in the protein database [17], we found many orthologs of Cropped throughout the animal kingdom. The AP-4 protein sequences of 39 different species of animals with their genomes sequenced were compared and aligned according to the similarity in the protein sequences using the multiple sequence alignment CLUSTALW program developed by the Kyoto University Bioinformatics Center. Based upon their similarity in amino acid sequence, a rooted phylogenetic tree was built on the found AP-4 orthologs (Additional file 2: Figure S1), and the proteins can be divided into 2 major clades: Group A, consisting of many species of insects, including all of the *Drosophila* species sequenced as well as *Lepeophtheirus salmonis* (the sea louse, a Copepoda) and *Trichoplax adhaerens* (Phylum Placozoa); and Group B, a more heterogeneous group encompassing the snail, *Schistosoma mansoni*, the sea squirt, and many species of mammals. Among the members with each clade, they have high homology within their conserved domains. All AP-4 proteins contain 4 highly conserved regions that are present in many species. These four highly conserved motifs are presented in Figure 2B and C and they are the basic-helix-loop-helix (bHLH) domain for DNA-binding and dimerization, which is highly conserved among 39 species but seven. Two conserved leucine zipper domains are present for homodimerization of the protein [18].

*Drosophila* AP-4 protein has been characterized biochemically [19]. There are two isoforms, a major ~86-kD species and a minor ~78-kD species, the latter of which may result from translation starting with an internal methionine initiation codon. We raised polyclonal rabbit antisera against the recombinant Cropped protein (starting from residues 65 to 631), an N-terminal region (residues 65 to 289), and a synthetic peptide representing residues 176 to 190. All three of the antisera recognized a major band (“L”, ~86 kD) in immunoblots of extracts of embryonic and larval tissues (Figure 2D and data not shown). This band was absent in the extracts of *crp*^*35Fd2*^ mutant embryos (Figure 2D, lane 2). The minor, lower-molecular-weight species (“S”, ~78 kD) co-migrated with an engineered N-terminal truncation of Cropped (Cropped^S^) that derives from an alternative initiation site expressed under the heat-shock promoter (Figure 2E) and that has the same size when it is expressed in a recombinant form in *E. coli* (Figure 2F).

### Cropped is broadly expressed and localized to both the cytoplasm and nuclei in many larval tissues

The expression pattern of *crp* in the larva was determined by the *in situ* hybridization of dissected third-instar larval tissues and the analysis of *crp P[lacZ]* enhancer-trap markers (Additional file 1: Table S1). The *cropped* mRNA was broadly expressed in the larva (Additional file 2: Figure S2). This transcript was expressed at low levels throughout many larval tissues, including the muscles, the fat body, the tracheal system and the epidermis, and at much higher levels in the central nervous system (CNS), the endodermal cells of the gut, heart, and many imaginal tissues, (Additional file 2: Figure S2A-S2D and data not shown). The *crp*-*lacZ* markers *k00232* and *k03101* showed similar patterns of expression (Additional file 1: Table S1). *crp* mRNA was expressed broadly and at a high level in the CNS at the end of embryogenesis (Additional file 2: Figure S2E-S2H). Previously, a developmental microarray analysis shows that *cropped* RNA is expressed throughout development, including the pupal and adult stages [20]. The immunostaining of larval tissues with the two antisera against the Cropped protein showed that this protein was also broadly expressed, with elevated levels of expression in the same tissues as for the *cropped* mRNA. As with the mRNA of *crp*, high expression of Cropped was found in many tissues (Additional file 2: Figure S3) suggesting that Cropped may be required for general functions other than tracheal branching (see below).

### The expression levels of *crp* affect the branching of tracheal terminal cells

**Figure 3:**
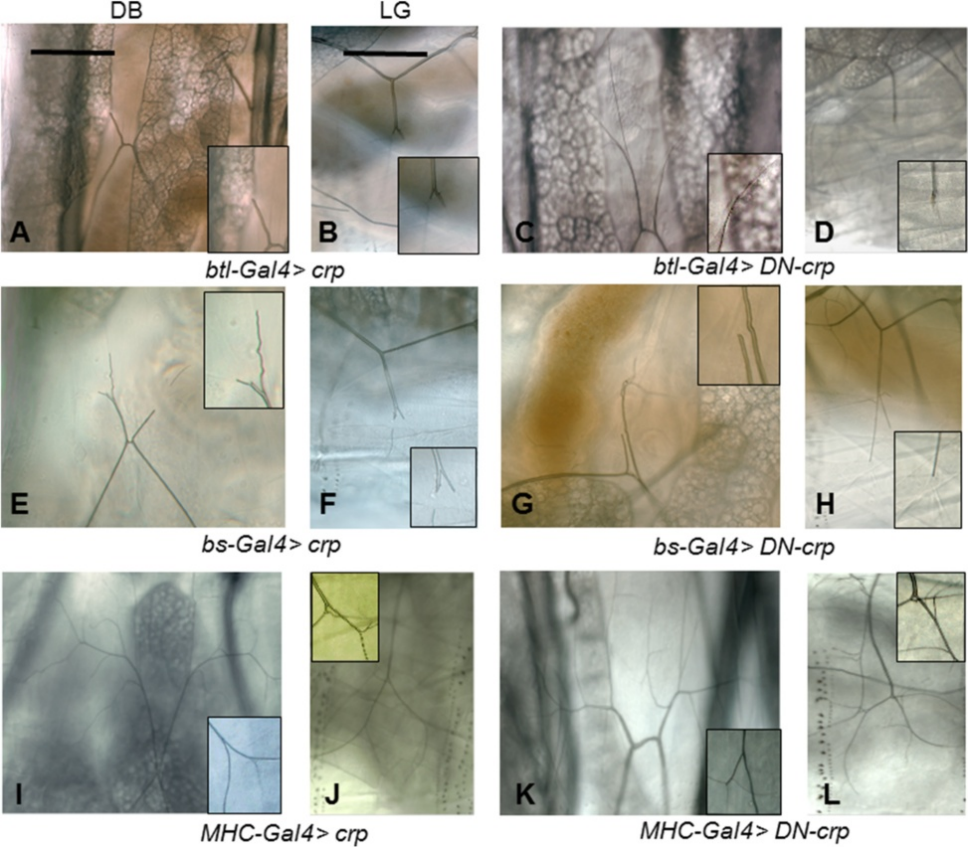
*crp* functions autonomously in tracheal terminal cells. *UAS-crp* transgenes that express the WT Cropped protein **(A, B, E, F, I, and J)** or DN-Cropped **(C, D, G, H, K, and L)** were expressed in the tracheal system by *btl-Gal4* **(A-D)**, terminal cells by *bs-Gal4* **(E-H)**, or muscles by *MHC-Gal4* **(I-L)**. The photomicrographs show DB and LG branches in third-instar larvae visualized under DIC optics. The insets show the terminal branches at higher magnification. The scale bar = 50 μm.

To determine whether *crp* functions in tracheal cells or its target tissues to promote terminal branching, we created transgenes that express a wild-type Cropped protein (by *UAS-crp*) and a dominant-negative (DN) form (*UAS*-*DN-crp*) lacking the DNA-binding domain. It has been demonstrated that the human AP-4 protein can interact with itself to form a dimer but is not able to dimerize with other closely related bHLH proteins, such as E14 [18]. Thus, it is likely that, in vivo, the DN mutant protein would dimerize with the endogenous Cropped to cripple the DNA binding ability of the complex. When *UAS-DN-crp* was specifically expressed in the whole tracheal system using *btl-Gal4* driver, the number of thicker terminal branches of DB from segment 7 was reduced to 2.93 ± 0.47 (n = 53) (Figure 3C) while that of wild-type was 6.30 ± 1.06 (n = 96). The average number of terminal branches in LG in wild-type larvae was 13.92 ± 0.46 (n = 72), whereas in *DN-crp* expressing larvae the number decreased to 6.85 ± 0.33 (n = 80) (Figure 3D). These observations demonstrate that *DN-crp* mimicked the *crp* loss-of-function phenotype. We also tried expressing *crp*-specific siRNA under the UAS control (the Fly stock numbers 31896 and 37470) with the same *bs*- and *btl-Gal4* drivers at several temperatures from 22 to 26°C, and unfortunately the progeny from these crosses did not show any tracheal branching phenotypes (data not shown).

On the other hand, quite unexpectedly, the overexpression of wild-type *cropped* (*UAS*-*crp*) using the same driver (Figure 3A-B) phenocopied the *crp* or *DN*-*crp* mutants. Because *btl-Gal4* directs the expression of *crp* in the whole tracheal system, which may affect terminal cells indirectly, we also employed the *bs-Gal4* line, which specifies the expression only to terminal cells and weakly in muscle cells. The phenotypes of the two *crp* transgenes were identical with those driven by *btl-Gal4* (Figure 3E-H), suggesting that *crp* functions mainly in terminal cells for cellular branching. Since either overexpression of wild-type *crp* or *DN-crp* resulted in no terminal branches, we would like to eliminate the possibility that the truncation in terminal branch is an inherent artifact of overexpression of proteins in terminal cells by the *Gal4-UAS* system. In all the progeny of the crosses with overexpressing destabilized red fluorescent protein (DsRed) and actin-GFP by the same driver, all terminal branches examine did not exhibit any phenotypes (Additional file 2: Figure S4). In contrast, when *crp* or *DN-crp* was expressed in the muscles using the *MHC*-*Gal4* driver (a muscle-specific driver), there was no reduction in the number of terminal branches (Figure 3I-L). We conclude that *crp* functions mainly in terminal cells to promote the outgrowth of terminal branches.

### Cropped is involved in controlling cell growth and size

**Figure 4:**
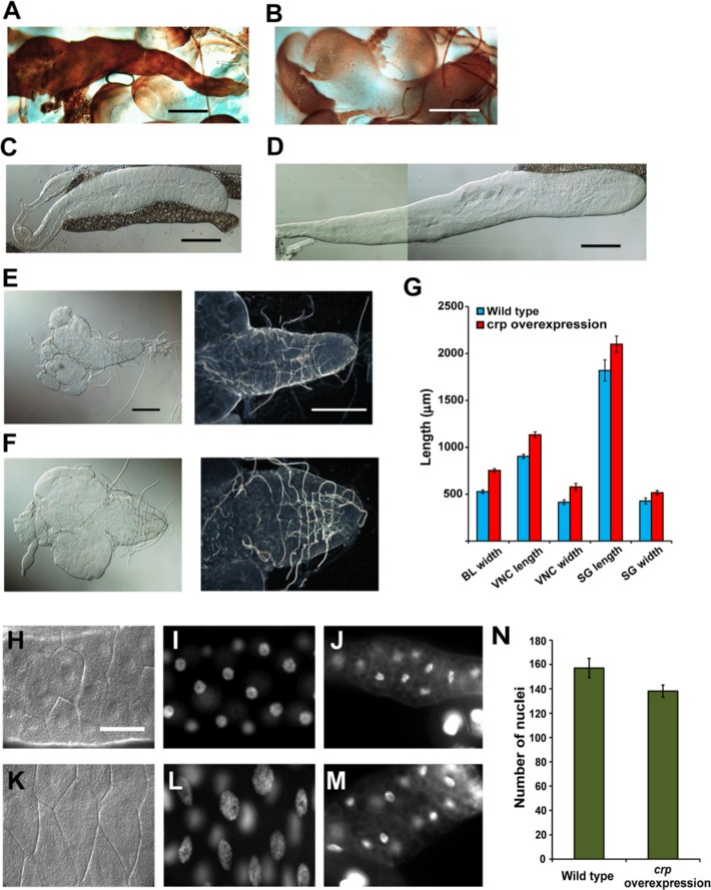
Cropped controls the sizes of organs and individual cells and not cell number in larvae. **(A)** The *cropped* transgene was expressed at high levels with *A9-Gal4* driver in many tissues in L3 larvae. The protein level of Cropped in the salivary gland **(A)** and nerve cord and imaginal discs **(B)** was visualized with the brown stain from horseradish peroxidase bound to the secondary antibody. The salivary glands from WT **(C)** and *A9-Gal4* overexpressing the *cropped* transgene **(D)** were dissected from the larvae. The central nervous system from WT **(E)** and overexpressing the *cropped* transgene **(F)** were also removed from the larvae for size comparison. The photomicrographs were taken with the same power of magnification, and the pictures on the right were using dark-field microscopy. **(G)** The length and width of the salivary glands, brain lobes, and ventral nerve cord were measured. The blue bars were the measurements from WT larvae, and the red bars were from Cropped-overexpressing larvae. The errors above the bars were SEM. The scale bar = 150 μm from **A** to **F**. From **H** to **M**, the salivary glands and fat body from both WT **(H-J)** and *A9-Gal4* overexpressing *cropped* **(K-M)** were stained with DAPI to identify the nuclei of the cells. The cells of the salivary glands from WT larvae **(H and I)** and from *Cropped*-overexpressing larvae **(K and L)** are shown under DIC optics **(H and K)** and DAPI-stained **(I and K)**. **J** and **M** are photomicrographs of DAPI-stained fat body cells. From H to K, the photographs were taken with the same magnification, and the scale bar indicates 60 μm. **(N)** The quantitation of the number of nuclei from the salivary glands of the two types of larvae.

As we tried to express the *crp* gene in various organs outside of the tracheal system with different *Gal4* drivers to examine whether changing the *crp* levels can affect the tracheal branching around these organs, we confirmed the levels of Cropped protein by immuno-histochemical staining with anti-Cropped antibody. It was expressed under the *A9-Gal* driver at very high levels in the salivary glands (Figure 4A), central nervous system (Figure 4B), and various other organs. Surprisingly, we found that the sizes of the salivary glands were larger than those from wild-type larvae (Figure 4C-D). The central nervous system of the larvae was also visibly bulkier in both the brain lobes and the ventral nerve cord (Figure 4F, E). To render the observation statistically reliable, twenty samples of salivary glands and CNS were dissected out, and digital photographs were taken for dimension measurement. The average longitudinal dimension of the salivary glands (distal end to the point between the gland proper and the stalk) from the from wild-type larvae was 1.85 ± 0.028 mm (N = 49) and that from *crp*-overexpressing larvae was 2.16 ± 0.049 mm (N = 50). The measurements showed that the length increased 27.7% and the width increased 21.6% in the larvae (Figure 4G). The diameter of the brain lobe increased by 40.4%, whereas the length of the ventral nerve cord increased by 25.6% and the width increased by 38.9%. These results suggest that an increased Cropped protein level increases the size of the organs between 20% to 40%. A similar degree of increase in organ size was reported in mutants of the insulin signaling pathway [21,22].

As exemplified by the various mutants of the insulin signaling pathway, increases in organ size in endoreplicating tissues, such as the tracheal cells, are primarily due to increases in cell size rather than cell number, but it was also possible that Cropped was affecting the cell number. To differentiate whether this gene affects cell size, cell number or both, the salivary glands dissected from *crp*-overexpressing and wild-type larvae were fixed and stained with 4′-6-diamidino-2-phenylindole (DAPI) to localize the nucleus of each cell (Figure 4H, I, K, L). The number of bright fluorescent nuclei in the salivary glands from 20 larvae was counted. The mean number of nuclei in the salivary glands of the wild-type larva was 157, whereas that of larvae with a high level of Cropped protein was 138 (Figure 4N). A similar increase in the size was observed in the cells of the fat body (Figure 4J, M). These results suggest that *crp* induces the formation of large salivary glands by increasing the cell size and not much affecting the cell number.

### Loss of Cropped DNA binding ability leads to inhibition of cell proliferation and increased apoptosis in the trachea and salivary glands

**Figure 5:**
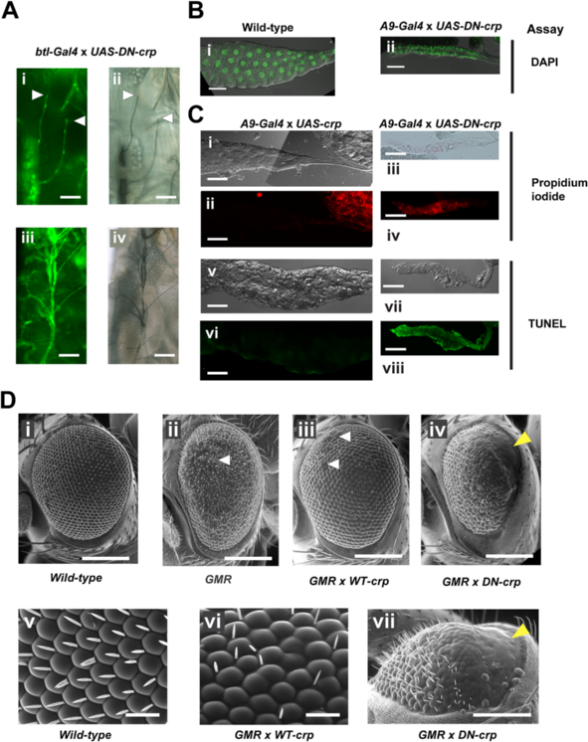
Expression of dominant-negative Cropped leads to missing cells and disruption of organ development. **(A)** Overexpressing *DN-crp* by using *btl-Gal4* in the tracheal system led to disruption of the formation of terminal and fusion cells (i and ii); the arrow heads point to the ends of DBs. (iii and iv) Larvae show a collapse in dorsal trunk. (i and iii) are fluorescence images of GFP-labeled tracheal cells and (ii and iv) are the same field under DIC optics. **(B)** Expression of *DN-crp* in the salivary glands driven by *A9-Gal4* shrank the organs (ii) compared with control (i) under the same magnification. **(C)** Staining assay on the cells expressing *crp* and *DN-crp* in the salivary glands for apoptosis with changes in permeability by PI (i-iv) and breakage of DNA strands by the TUNEL assay (v-viii). DIC photomicrographs (i, iii, v, vii) and fluorescence microscopy (ii, iv, vi, viii) were taken under the same magnification. **(D)** Scanning electron micrographs of the ommatidia expressing *DN-crp* or WT *crp* driven by *GMR*-*Gal4*. (i-iv) The anterior of the fly is pointing to the left and the posterior to the right. *DN-crp* inhibited the formation of ommatidia and bristles in the posterior half of the eyes and WT *crp* caused overgrowth of groups of ommatidia. The white arrowheads indicate groups of protruding ommatidia in (ii and iii) and the yellow arrowheads points to the posterior end of the eye without any ommatidia. Scanning micrographs at higher magnification of the ommatidia from WT (v), WT *crp*- (vi), and *DN-crp*-expressing fly (vii) are shown. (vii) Electron micrograph taken from the dorsal side of the eye of *DN-crp* expressing fly and the anterior is to the left. Scale bars are 100 μm **(A, B, and C)**, 200 μm (Di-iv), 20 μm (Dv,vi), and 50 μm (Dvii).

During the examination of tracheal branching phenotype of overexpressing *DN-crp* in the tracheal system with the *btl-Gal4* driver, we found that 62% (n = 34) of the L2 and L3 larvae displayed a very severe phenotype in which the dorsal trunks of the larvae were collapsed (Figure 5Ai-iv). With the aid of the fluorescence signal from the nuclear GFP expressed in the whole tracheal system, there were just 3 to 4 cells along DB and the last cells could still attach to the somatic muscles (white arrow heads in Figure 5Ai, ii), but no terminal cells and fusion cells were observed. This indicates that *DN-crp* expressed in the tracheal system may have adversely affected the normal development of DB.

When *DN-crp* was expressed in the salivary gland driven by *A9-Gal4*, the glands shrank into a much smaller and shorter organ (Figure 5Bii, average length = 0.586 ± 0.05 mm, n = 28) when compared with the wild-type salivary glands (Figure 5Bi, average length = 1.85 ± 0.028 mm, n = 49). The shortened salivary glands contained only 88 ± 4.30 cells (n = 25), while the wild-type glands contained 157 ± 8.21 cells (n = 31). It is obvious to observe that the sizes of individual cells in *DN-crp* expressing glands were much smaller than those of the wild-type and the same is true for the sizes of their nuclei (Figure 5Bii), indicating that *crp* may have affected cell growth and thus probably endoreplication. Moreover, the decrease in cell number in the glands suggests that *DN-crp* may inhibit the development of the glands by either slowing down the cell proliferation rate or by enhancing apoptosis of the cells.

To demonstrate whether cell viability plays a role in decreasing the number of cells in *crp-* and *DN-crp*-expressing salivary glands, we stained the freshly dissected salivary glands from the larvae with propidium iodide (PI) without any fixation to observe the presence of dying cells, which are permeable to PI. Most of the glands from *crp*-overexpressing larvae did not stain with PI (Figure 5Ci, ii) and all the glands from *DN-crp* expressing larvae were stained positively with PI (Figure 5Ciii-iv), suggesting that the smaller gland size may be due to the non-viability of the cells caused by overexpression of *DN-crp*. To confirm whether the decrease in viability is caused by apoptosis, the TUNEL assay was employed. Similar to the results of PI staining, only the salivary glands from *DN-crp* expressing larvae stained positively with the TUNEL reagent while *crp* expression did not give any signals (Figure 5Cv-viii). In addition, we stained these gland cells with cleaved caspase-3 antibody to examine whether the caspase-3 dependent apoptotic pathway is activated. The signals from the antibody staining in both *crp*- and *DN-crp* expressing gland cells were all negative (data not shown), suggesting that *DN-crp* expression caused suppression in both cell growth and number by increasing apoptosis in a caspase-3 independent manner.

To examine whether *crp* expression affects eye development, ectopic expression of *DN-crp* driven by *GMR-Gal4* in the eye imaginal discs led to a gradient of defects of the ommatidia and bristles on the posterior half of the adult eyes (yellow arrow head in Figure 5Div, vii). The most posterior end of the eyes bore no ommatidia and bristles, whilst closer to the midline only ommatidia were found without any bristles. The anterior side of the eyes contained both round ommatidia and normal bristles as wild-type (Figure 5Dvii vs. 5Dv). In contrast, the pattern of ommatidia from flies bearing overexpressing *crp* was slightly disorganized, and each ommatidium appeared slightly more bulky than wild-type (Figure 5Diii, vi). Occasionally, groups of them appeared more protruding in the dorsal half of the eyes of every adult flies (n = 21) (white arrow heads in Figure 5Diii) and, intriguingly, the growth of bristles associated within the eyes was suppressed (Figure 5Dvi). The results from these experiments show that inhibition of the transcriptional activity of Cropped (using *DN-crp*) resulted in the loss of cells in many organs, including terminal cells, salivary glands, and the eyes. However, the outcomes of *crp* overexpression would depend on the cell type or tissue; it inhibited the differentiation of terminal cells and the eye bristles, but it induced excessive cell growth of the salivary glands and ommatidia.

### dMyc activates *crp* expression and induces terminal branching

**Figure 6:**
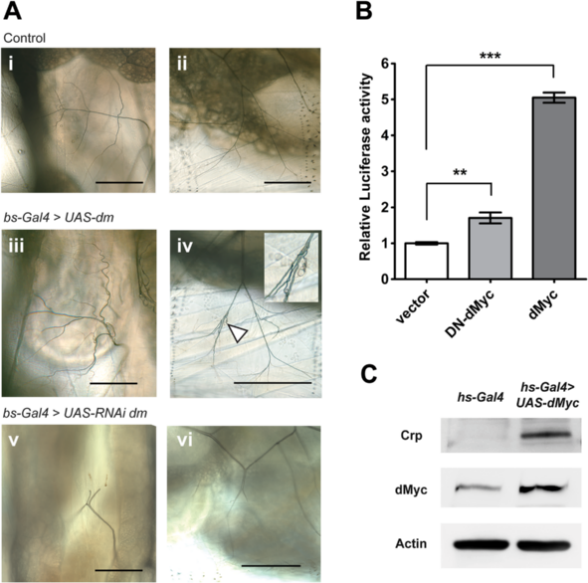
The induction of dMyc (dm) increases terminal branching and activates *crp* expression. **(A)** The terminal branches of DB (i, iii, and v) and LG (ii, iv, and vi) of L3 larvae were observed. Ectopic expression of dMyc in terminal cells with *bs-Gal4* resulted in the formation of excessive and tortuous terminal branches of the DB (iii) and LG (iv) compared with those of larvae without *UAS-dm* (i and ii). Depletion of endogenous c-MYC by expressing siRNA against *dm* induced truncation of both DB (v) and LG (vi). The scale bar indicates 200 μm. **(B)** dMyc induced *crp* promoter activity. S2 cells transfected with the plasmid expressing firefly luciferase gene downstream of the *crp* promoter fragment, the plasmid expressing dMyc or dMyc without the DNA binding region (DN-dMyc), and the Renilla luciferase expressing plasmid (as transfection control) for 48 hours and then harvested for reporter gene assays. The ratio of firefly luciferase to Renilla luciferase luminescence signal was calculated and fold changes of luciferase activity relative to vector control are shown. Values are represented as mean ± SEM from four independent experiments. ** indicates *p* value <0.01, *** indicates *p* value <0.001. **(C)** Overexpression of dMyc in larvae with the genotype *hs-Gal4;* *UAS-dMyc* was observed after exposing the larvae to 35°C for 20 min. Crp, dMyc, and actin were visualized by Western blots using their specific antibodies.

Recently, it has been demonstrated that, in mammals, the *AP-4* gene is regulated by c-Myc [23]. We hypothesized that this regulatory hierarchy may be conserved in *Drosophila*. It has been shown that, in *dm* (encoding dMyc) mutants*,* both the cells and the organisms are smaller than the wild-type, whereas dMyc overexpression increases cell size [24]. Therefore, we assumed that if *dm* acts upstream of *crp*, then changes in *dm* expression should have a tracheal branching phenotype. We tested this notion by overexpressing *dm* in terminal cells with *bs-Gal4* and observing any morphological changes in tracheal branching. The overexpression of *dm* in terminal cells did not increase the number of thicker terminal branches but instead it increased the formation of fine terminal branches (indicated by white arrow head, Figure 6A) of DB (Figure 6Aiii) and LG (Figure 6Aiv) branches compared with the *bs-Gal4* strain (Figure 6Ai-ii). However, the increase in the formation of short, fine branches occurred mainly near the terminal cell body (insert of Figure 6Aiv and Additional file 2: Figure S5). In addition, some of the thick terminal branches appeared more tortuous and the air-filled lumens of these branches were not even in diameter (uneven dilatation of the lumen) (Additional file 2: Figure S5Aiii and S5Biii), which are similar to the effects of overexpressing *bnl* or of hypoxia [6,7]. In contrast, the expression of short interfering double-strand RNA of *dm* in terminal cells by *bs*-Gal4 resulted in the truncation of terminal branches similar to that of the *crp* mutants and *DN-crp*- or *crp*-overexpressing larvae (Figure 6Av-vi).

To examine whether *dm* can regulate *crp* expression and tracheal branching, the genomic DNA sequences of *crp* from 6 different species of *Drosophila*, including *D. melanogaster*, *D. simulans*, *D. yakuba*, *D. erecta*, *D. virilis*, and *D. pseudoobscura,* were aligned using ClustalW, and the E-boxes of both Myc and AP-4 were located (Additional file 2: Figure S6). A single canonical c-Myc-binding site (CACGTG) was present at 55 nucleotides upstream of the translation start site (ATG) of the *crp* gene in four species of *Drosophila*, whereas the dMyc binding site was located 89 and 100 nucleotides upstream from the ATG site in *D. virilis* and *D. pseudoobscura*, respectively. The conservation of the dMyc binding site in different species suggests that *dm* may be able to regulate *crp* expression.

To demonstrate that dMyc can transactivate *crp*, we transfected the plasmid expressing the *dmyc* gene under the control of the actin 5C promoter, pGL4.10 plasmid containing the promoter sequence (626 bp DNA sequence containing Myc binding site, indicated as blue brackets in Additional file 2: Figure S6) of the *crp* gene cloned upstream of the firefly luciferase gene, and the Renilla luciferase expressing plasmid, pAc5.1C-RLuc-V5-His6, into *Drosophila* S2 cells. The Renilla luciferase activity was used for the normalization of transfection efficiency. When dMyc was expressed, the relative luciferase activity was 5-fold higher compared than control (without dMyc expressed), while dominant-negative dMyc (dMyc without the DNA binding region) only slightly induced the luciferase activity from the same promoter (Figure 6B). The high induction of *crp* promoter activity was observed over the control transfection, suggesting that dMyc is a strong inducer of the *crp* gene transcriptional activity.

To observe whether the same transcriptional relationship between dMyc and *crp* in vivo, we induced the expression of dMyc in L3 larvae harboring both *heat shock-Gal4 (hs-Gal4)* and *UAS-dm* transgenes by heating at 35°C for 20 minutes. The increase in dMyc protein expression after the heat shock resulted in the induction of Cropped protein, as visualized by Western blotting (Figure 6C). These experiments suggest that dMyc functions similarly to its mammalian counterpart in activating the expression of *crp* to control tracheal branching.

## Discussion

In the present study, we used an enhancer-trap genetic screen to find novel genes that are involved in the development of tracheal terminal branching at the larval stage of *Drosophila*. In this paper, we described one of the three mutants that exhibit truncated terminal branches, the *cropped* mutant. Molecular cloning and analysis showed that the mutated gene, *cropped,* encodes the *Drosophila* homolog of the mammalian transcription factor AP-4. The human AP-4 protein was discovered by its ability to bind to the long terminal repeat (LTR) sequences of the SV40 genome [25]. Recently, it has been implicated in cell proliferation [23], but its molecular functions remain largely elusive.

Results from biochemical experiments demonstrated that the human AP-4 protein can only form homodimers via the two leucine zipper domains and that it binds to the E-box sequence [18]. The nucleotide sequence bound by Cropped in *Drosophila* has been shown to be identical to the human counterpart [19]. In the literature, there are a myriad of genes that are decorated with AP-4 binding sequences, both upstream of promoters and within introns; however, only a few studies have demonstrated the direct binding of AP-4 to the sequence and the functions of the genes involved. The mammalian AP-4 has been described, in certain cases, as an activating factor [25], and as a repressor in other physiological contexts [26-28]. The only protein known to interact with Cropped/AP-4 is Daughterless, which is also an HLH protein, but the physiological significance of the interaction between Daughterless and Cropped in the salivary gland is not clear [19].

### Cropped is necessary for tracheal terminal branching

In the present study, we showed that Cropped is necessary for the formation of tracheal terminal branches in both the embryonic and larval stages. To inactivate the functions of the endogenous AP-4 protein, we expressed *DN-crp* in various tissues to elucidate its functions in vivo. When either WT or *DN-crp* was expressed in the tracheal tissues, the formation of terminal branches was impeded, but this effect was not observed for expression in the muscles, suggesting that *crp* acts mainly in the tracheal tissues for tracheal branching. The cytoplasmic extensions of the terminal branches, as visualized by the presence of GFP expressed in the tracheal tissues, were much less extensive but still present. The air-filled lumen in the terminal branches extended only slightly beyond the locations of the terminal cell bodies. Moreover, the number of viable homozygous *cropped* mutant larvae is less than expected, and the homozygotes seldom develop into flying adults, implying that *crp* mutants may be lethal due to the impairment of gas exchange or probably cell growth as discussed below.

### Functions of the Cropped protein outside the tracheal system

The requirement of the Cropped protein only in the trachea cells for the formation of the tracheal terminal branches appears to contradict our observation that both the protein and mRNA expression of Cropped are ubiquitous in both embryonic and larval bodies. Because the expression patterns of Cropped and Branchless are very similar, we expected that overexpression of *crp* in tissues other than the trachea (in the muscles, for example) might increase branching by stimulating Branchless expression. However, we did not observe any changes in branching and coverage. Instead, an increase in the size of the salivary glands and central nervous system was observed by overexpression of WT *crp*. In addition to an obvious increase in cell dimensions, the quantification of the cell number in the salivary glands suggests that Cropped controls the cell size but not the cell number. This observation is consistent with the finding in a study that shows that dAP-4 regulates cell size using a genome-wide RNAi methodology [29].

It has recently been demonstrated that mammalian AP-4 is involved in cell proliferation and is under the control of Myc [23]. This report is consistent with the finding that dMyc can also affect cell growth and proliferation in *Drosophila*. Similar to c-Myc, the increase in size of the cell and nucleus in Cropped-overexpressing tissues suggests that dAP-4 may be involved in endoreplication, in which the cells grow without undergoing mitosis or cytokinesis [30]. Looking for evidence that *crp* is controlled by dMyc, as in mammals AP-4 is controlled by c-Myc, we found that there is a strong conservation of a single Myc-binding sequence, CACGTG, upstream of the translation start site (−55 bp from the ATG) in 4 species of *Drosophila*. Overexpression of dMyc in *Drosophila* S2 cells enhanced 5-fold the relative promoter activity of *crp* compared with control , showing that dMyc can indeed drive the expression of *crp*. Since dMyc has been shown to increase cell size in vivo and Cropped controls both cell growth and tracheal branching, we hypothesized that dMyc would also increase tracheal branching if dMyc acts upstream of *crp* to increase cell growth. Indeed, higher levels of dMyc in terminal cells resulted in an increase in the number and tortuosity of tracheal branches, similar to that caused by the overexpression of *bnl* or after hypoxic exposure [6,7]. In addition, the overexpression of dMyc protein induced the expression of *cropped*. These experiments support the notion that Cropped/AP-4 is a downstream target of dMyc and that both of these proteins are involved in cell growth and tracheal branching in *Drosophila*.

### A link between cell growth and tracheal branching

It was initially quite perplexing how Cropped controls tracheal terminal branching and controls another seemingly unrelated process, cell growth. The answer would be more apparent if one considers that the numerous terminal outgrowths in each branch are originated from a single terminal cell. Each terminal cell only harbors a single, short terminal branch in embryo and ramifies the branch to numerous cytoplasmic extensions in all different directions on the tissues they contact during the larval stage. Terminal cells need to increase not only the surface for gaseous exchange but also its own cell size to cover the target tissues. As we found that the *cropped* gene and Cropped protein are expressed at high levels in many tissues including the nervous tissues during both embryogenesis and the larval stages, we hypothesize that other cell types, such as neurons and endothelial cells, may utilize a similar process in increasing the cell size and cellular content of the growth cone to extend towards their targets.

This process could be visualized as a case of the simplification and coordination of molecular functions, in which Cropped protein acts as both a cell type-specific transcription factor in controlling tracheal terminal branching and as an activator of cell growth under the control of the same signaling mechanism. With an ample supply of nutrients, such as glucose and amino acids, many endocycling tissues in larvae undergo cell growth [22]. Branchless/FGF activates the MAPK pathway, which induces the Cropped protein in many tissues to grow in cell size. At the same time, the cell growth mechanism mediated by the dMyc-Crp axis also occurs in terminal cells to increase in cellular branches. By this argument, the control of the two different physiological processes, cell growth and branching, can be coordinated by the same signaling mechanism in terminal cells.

Angiogenesis in mammals is a process that is analogous to insect tracheogenesis in function and molecular mechanism [31]; therefore, we speculate that the mammalian AP-4 protein may function in a similar fashion in angiogenesis. During angiogenesis, new blood vessels branch out onto hypoxic target tissues due to the proliferation and migration of the tip endothelial cells via the attraction of angiogenic chemoattractants, such as VEGF or FGF [32]. The growth of the endothelial cells towards the target tissues involves cell division and cell growth of the tip cells, but the insect tracheal terminal cells increase in cell size by endoreplication to attain full coverage of the oxygen-starved target tissues.

## Conclusions

Transcription factor AP-4 or Cropped is expressed ubiquitously in almost every cell in the larvae of *Drosophila*. The functions of the *cropped* gene in terminal cells of the tracheal system are regulating cell growth and cellular branching. Cropped may also affect the development of the ommatidia and salivary glands in their cell size and numbers depending on the levels of Cropped expression.

## Methods

### Plasmids

pGL4.10-*crp*-pro was constructed by PCR amplifying the promoter region of the *Drosophila crp* gene (forward primer is 5′ CTGGTACCATCGCAGTGGCATCAATGT 3′ and reverse primer is 5′ CTGGTACCGCGGACATGTTTAATCGTG 3′) and cloned into pGL4.10 vector (Promega Madison, WI). pAc5.1C-RLuc plasmid was purchased from Addgene (# 21182). pAc5.1C-*dmyc* was constructed by the PCR-amplified *dmyc* gene from a transgenic fly carrying a wild-type copy of *diminutive* (*dm*), *dmyc* (Bloomington stock number 9875) with forward primer 5′ CTGCAGAATTCGCTATGGCCCTTTACCGCTCT 3′ and reverse primer 5′ GTTAGGGATCCTCCACTAACCGAGCGCGATT 3′, and cloned into the pAc5.1C vector (# V4110-20, Invitrogen, Carlsbad, CA). pAc5.1C-DN-*dmyc* was constructed by PCR amplifying the whole pAc5.1C-*dmyc* plasmid (forward primer 5′ ATTGGACTAAAGAACCTCTTTGAG 3′ and reverse primer 5′ GATCGTATCGGCCTCATCA 3′) without including the DNA binding region (the DNA sequence corresponding to EKRNQHNDMERQRR) of dMyc. The sequences of all constructed plasmids were confirmed by DNA sequencing.

### Fly strains and genetics

The *cropped* alleles *l(2)k00232, l(2)k10415, l(2)k00809, l(2)k7829, l(2)k13611, l(2)k05601,* and *l(2)35Fd2/RAR46* have been described [33,34]. The alleles *l(2)35Dh[AS64]* and *l(2)35Di[RAR8]* are linked to mutations in other genes generated in the same mutagenesis as *l(2)35Fd2/RAR46*. The allele *blistered* (*pruned*)^*ex84*^ is a null mutation [8]. *Df(2 L)r10* and *Df(2 L)RN10* carry deletions of the cytological regions 35E1-36A7 and 35E1-36A5, respectively, including the *crp* locus. Canton-S was the wild-type strain.

The tissue-specific Gal4 driver lines *btl-Gal4* (embryonic and larval trachea) [35], *5053A-Gal4* (somatic muscles in late embryo and larva), *MHC-Gal4* (larval somatic muscles), and *A9-Gal4* (larval central nervous system, salivary gland and others) have been described. A *btl-Gal4.UAS-GFP* chromosome (constructed by D. Micklem and M. Metzstein), which drives the expression of GFP in the tracheal nuclei and cytoplasm, was used to visualize the cytoplasmic extensions of the developing terminal branches. *bs-Gal4* is used to express protein in terminal cells and somatic muscles in larvae. The following UAS and Gal4 lines were used for ectopic expression: *UAS-crp*, *UAS-DN-crp* (this work), *UAS-dm*, *UAS-RNAi-dm*, *UAS-DsRed*, *UAS-actin*, *btl-Gal4* [35], *bs-Gal4* [36], *MHC-Gal4*, *A9-Gal4*, and *GMR-Gal4*.

The original *crp* allele, *l(2)k10415,* was identified in a screen of 250 P[*lacZ, w+*] insertions on the second chromosome [37] for larval tracheal defects. A CyO Tb balancer chromosome was placed *in trans* to each mutation. The non-Tb larvae were collected, washed with PBS, and immobilized by immersion in a solution of 0.5% sodium azide. After 20 minutes, the larvae were placed on glass slides and covered with 50% glycerol. The tracheal morphology was examined under differential interference contrast (DIC) optics with a Zeiss Axioskop (Zeiss, Jena, Germany).

### Molecular biology

The full-length *crp* cDNA in pBluescriptSK+ was provided by Drs. Ling Hong and Gerry Rubin (University of California, Berkeley). The cDNA insert was sequenced on both strands by dideoxy chain-terminator sequencing and analyzed on an ABI 310 capillary electrophoresis DNA sequencer. The following online programs were used for the analysis, alignment, and identification of domains in the sequenced cDNA: NCBI BLAST homology searches, BEAUTY post-processing (Human Genome Sequencing Center, Baylor College of Medicine), CLUSTAL W multiple sequence alignment (1.81) (Kyoto University Bioinformatics Center) and the COILS program.

To identify the *crp*^*35Fd2*^ mutation, the exons of the *crp* locus were PCR-amplified from genomic DNA isolated from *crp*^*35Fd2*^*/CyO* adult flies and sequenced on both strands. A single coding change was identified and confirmed in three independent PCR products. The insertion sites of *crp P[lacZ, w+]* were identified by plasmid rescues from *E. coli,* and DNA sequencing was performed to identify the junctions between *P[lacZ,w+]* and the flanking genomic DNA.

### Overexpression of *cropped* on organ size in the central nervous system and salivary glands

Flies carrying a wild-type *crp* gene (*UAS*-*crp*) were crossed with flies with *A9-Gal4*. The larvae from the crosses were dissected and stained histochemically with an anti-Cropped antibody to observe the overexpression of the Cropped protein in different larval organs. For the measurement experiments, the salivary glands and the central nervous system from 20 larvae were carefully dissected out with forceps and transferred to several microscopic slides. The tissue was placed on glass slides and covered with 50% glycerol. Digital pictures were taken of all of the dissected organs, and their sizes in several dimensions were measured with rulers. For the experiment counting the number of cells in an organ, 20 salivary glands were dissected and fixed in 3.7% formaldehyde for 10 minutes before being stained with DAPI, which specifically binds to DNA and fluoresces under ultra-violet light illumination. The numbers of cells in the whole organs were counted under UV illumination with a counter.

### Transfection and luciferase assay

*Drosophila* Schneider 2 (S2) cells were propagated in Schneider’s *Drosophila* medium supplemented with 10% heat-inactivated FBS. Cellfectin II reagent (Invitrogen, Carlsbad, CA) was used for transfection according to the manufacturer’s instructions. In brief, 3 × 10^5^ S2 cells were seeded in a 24-well plate in unsupplemented Grace’s insect medium (SFM) for 2 hours before transfection. 80 ng of pAc5.1C-RLuc, 320 ng of pGL4.10-*crp*-pro, 100 ng of pAc5.1C-*dmyc*, pAc5.1C-DN-*dmyc* or empty pAc5.1C vector were diluted into 25 μl of SFM. Total amount of DNA per transfection was 500 ng. Two microliters of Cellfectin II was diluted into 25 μl of SFM, combined with the DNA mixture and incubated for 20 minutes at room temperature. The lipid-DNA complexes were added to the cells, incubated for 5 hours in a 28°C incubator and then replaced with fresh Schneider’s *Drosophila* medium with 10% heat-inactivated FBS. The cells were further incubated for 43 hours in the 28°C incubator. Luciferase activities were analyzed with the dual luciferase reporter assay (Promega Madison, WI), according to the manufacturer’s instructions.

### Crossing of flies

For terminal cell-specific expression of dsRed proteins, *UAS-DsRed-2 Nuclei* (II) was crossed with virgins of fly with either the *bs-Gal4* or *btl-Gal4* driver. The L3 larvae from the cross incubated at 26°C were removed for the examination of fluorescence signals in terminal branches under an Eclipse 80i fluorescent digital microscope (Nikon Instruments, Melville, NY).

### Immunostaining of embryos and larval tissues

The embryos were fixed and stained with antibodies (mAb2A12, 1:5 dilution; mAb2-161 against DSRF, 1:500; anti-β-gal, 1:500; anti-Cropped antisera, see below) as described [38,39]. For the staining of larval tissues, the larvae were pinned on a silicone elastomer platform and dissected along the ventral midline. The tissues were fixed in 3.7% formaldehyde for 10 minutes, pre-incubated with PBS containing 3% fetal calf serum for 30 minutes, and stained with anti-Cropped antiserum (1:1000 dilution) for 1–2 hours, followed by visualization using horseradish peroxidase immunohistochemistry (Vectastain ABC, Vector Labs, Burlingame, CA). Digital images were acquired with a Zeiss Axiophot microscope under DIC optics.

### *In situ* hybridization

The *in situ* hybridization of wild-type whole-mount embryos was performed as described [40], using a single-stranded digoxigenin-labeled *crp* cDNA probe. For the analysis of larval tissues, L3 larvae were dissected open along the ventral midline (see above), and most of the fat body was removed. The tissues were fixed in 3.7% formaldehyde for 10 minutes, and *in situ* hybridization was carried out as described above. The *crp* sense probe gave the expression patterns described in the text. The control experiments using an antisense *crp* probe in both embryos and larvae gave no detectable signal.

### Cropped antisera and immunoblotting

The DNA encoding Cropped residues 65 - 631 (Cropped^S^) or residues 65–289 (N-terminal) was amplified by PCR and cloned into the pET23a expression vector (Novagen). Expression in *E. coli* was induced by IPTG, and the histidine-tagged proteins were purified from the cell lysates by NTA affinity chromatography under denaturing conditions. The purified His-tagged proteins were injected into rabbits (Josman Labs, Napa, CA). Both antisera were used at 1:1000 for immunostaining and immunoblots. An anti-peptide antiserum was prepared by synthesizing a 16-residue peptide corresponding to Cropped residues 176 to 191, coupling it to Multiple Antigenic Peptide (MAP), and injecting it into rabbits (Research Genetics, Huntsville, AL). The peptide antiserum was purified by passing through a column of NHS-Sepharose coupled to the 16-residue Cropped peptide. The affinity-purified serum was used at a 1:500 dilution. The Cropped^S^ antiserum was used in all experiments shown, although all three antisera gave similar results on immunostaining and detected the 86 and 78 kD bands on immunoblots.

For the immunoblot analysis, *Drosophila* larvae and adults were dounce-homogenized in RIPA buffer (150 mM NaCl, 50 mM Tris–HCl (pH 8), 1% NP-40, 0.5% sodium deoxycholate, and 0.1% SDS). The protein concentrations of the homogenates were determined by a Bradford assay’. The extracts were boiled for 10 minutes in Laemmli sample buffer and clarified by centrifugation. One larval or adult equivalent of protein extract was separated by SDS-PAGE and transferred to nitrocellulose. The blots were stained with Ponceau S to confirm equal protein loading and transfer. The Cropped protein was detected with an anti-Cropped^S^ antiserum and HRP/chemiluminescent immunohistochemistry (ECL from GE Healthcare, Little Chalfont, Buckinghamshire, UK). The dMyc protein and actin were visualized with antibodies against dMyc (d1-717) (Santa Cruz Biotechnology, Santa Cruz, CA) and β-actin (Cell signaling Technology, Beverly, MA), respectively. For the immunoblot analysis of the recombinant Cropped protein, *E. coli* transfected with the pET23a-Cropped expression vector was grown to mid-log phase and harvested (without IPTG) or induced with IPTG for 1 hr (+IPTG). The cells were resuspended in Laemmli sample buffer and boiled for 2 min, and the equivalent denatured protein extracts were separated by SDS-PAGE.

### Ectopic expression of wild-type and dominant-negative Cropped proteins

The *GAL4/UAS* system [41] was used to express the wild-type and dominant-negative versions of the *crp* gene in different tissues. The vector *pUAS-crp* was constructed by inserting the full-length *crp* cDNA (*cropped*^*L*^) into the P-element vector *pUAST*. To construct *pUAS-DN-crp,* the DNA sequence corresponding to the basic region of the Cropped bHLH domain (K94 through R108) was deleted in the pBluescriptSK+ vector by inverse PCR, and the region flanking the deletion was exchanged with the corresponding region in *pUAS*-*crp*. The coding regions in *pUAS*-*crp* and *pUAS-DN-crp* were confirmed by DNA sequencing. P-element mediated transformation was used to obtain insertions of each transgene on the second and third chromosomes, and homozygous viable insertions on the second (*UAS*-*crp*-*168*) and third chromosomes (*UAS-DN-crp*-*238* and *UAS*-*crp*-*138*) were used. To persistently overexpress the wild-type or dominant-negative version of *Cropped* in the tracheal tissues in larvae, flies carrying the *UAS*-*crp* transgene or *UAS*-*DN-crp-238* were crossed with flies carrying *btl-Gal4UAS-crp-138* gave the same result as *UAS-crp-168*. In the control experiments, *5053A.-Gal4* was shown to drive a high level of expression of a *UAS*-*GFP* transgene in the muscles.

## Additional files

Additional file 1: Table S1. Expression of the *crp* gene and Crp protein in different tissues in L3 larvae. In all study here the larvae of wild-type (WT) and various *crp* mutants from the Kiss collection were dissected and individual organs were freed from other tissues before subjecting to staining with various methods. Additional file 2:
**This file contains Figures S1, S2, S3, S4, S5, and S6.** All supplementary figures and their legends are contained within this file.

## Abbreviations

AP-4: Activator protein 4
bHLH: Basic-helix-loop-helix
*bnl*: *branchless*
*btl*: *breathless*
*crp*: *cropped*
DAPI: 4′-6-diamidino-2-phenylindole
DB: Dorsal branch
DIC: Differential interference contrast
*dm*: *diminutive*
DN: Dominant-negative
LG: Lateral ganglionic
LZ: Leucine zipper
PI: Propidium iodide
WT: Wild-type
