# Supplementary material for: Cropped, Drosophila transcription factor AP-4, controls tracheal terminal branching and cell growth
Source: BMC Dev Biol. 2015 Apr 15;15:20. doi: 10.1186/s12861-015-0069-6 (PMC4430030; doi:10.1186/s12861-015-0069-6)
Supplement: Additional file 1: Table S1. — Expression of the crp gene and Crp protein in different tissues in L3 larvae. In all study here the larvae of wild-type (WT) and various crp mutants from the Kiss collection were dissected and individual organs were freed from other tissues before subjecting to staining with various methods. [file 12861_2015_69_MOESM1_ESM.pdf]

Supplementary table legend

Table S1. Expression of the *crp* gene and Crp protein in different tissues in L3 larvae

In all study here the larvae of wild-type (WT) and various *crp* mutants from the Kiss collection were dissected and individual organs were freed from other tissues before subjecting to staining with various methods. The expression of mRNA of *crp* was visualized in wild type larvae by in situ hybridization of the antisense strand probe of the *crp* gene. β-galactosidase activity conferred by the transposon in the heterozygous *crp* mutants from the Kiss collection was visualized with X-gal staining. Crp protein expression was visualized by staining with both antibodies specific to the N-terminal and the full length proteins. The staining pattern is referred to high when the color of the X-gal was stained dark blue specified in the staining period, medium when the color was lighter and low when the color intensity was just above the background. ND means not determined.

Table S1

Method	Strains	CNS	Imaginal disks	Pericardial cells	Fat body	Salivary gland	Trachea
**In situ**	WT	High	High	Medium	Very low	Very low / high, variable	Very low (variable)
**X-gal**	k00232	High	High	ND	No	ND	Medium
	k03101	High	High	High	No	High	Low
	k10415	High	High in antennal disc	Medium	No	High	Low
	k07829	High	High	High	High	High	High
**Protein**	N-term	High	High	Medium	Low	Variable but mostly low (some high)	High (N) anterior, low (C) posterior
	Full-length	High	High	Medium	Low	Variable but mostly low	High (N) anterior, low (C) posterior

Method	Strains	Macrophage	Epidermis	Muscle	Gut	Gastric caeca	Malpighian tubules
**In situ**	WT	Low	Low	Very low (variable)	High in some segments	Medium	ND
**X-gal**	k00232	ND	Medium	Low	High expression in a very limited region in midgut	ND	ND
	k03101	ND	Low	Low	High expression in a very limited region in midgut	ND	ND
	k10415	No	No	High	Midgut and hindgut boundary	ND	Low
	k07829	High	High	High	High	ND	ND
**Protein**	N-term	Medium	Low	Low (variable)	Some segments high	High	High
	Full-length	ND	Low	Low (variable)	Some segments high	High	High
