# Supplementary material for: Cropped, Drosophila transcription factor AP-4, controls tracheal terminal branching and cell growth
Source: BMC Dev Biol. 2015 Apr 15;15:20. doi: 10.1186/s12861-015-0069-6 (PMC4430030; doi:10.1186/s12861-015-0069-6)
Supplement: Additional file 2: — This file contains Figures S1, S2, S3, S4, S5, and S6. All supplementary figures and their legends are contained within this file. [file 12861_2015_69_MOESM2_ESM.pdf]

Supplementary figure legends

Figure S1. A phylogenetic tree showing the similarity in amino acid sequence of the AP-4 proteins in many animals

A phylogenetic tree generated from the ClustalW analysis on AP-4 proteins of various species of animals from the protein databases. The protein sequences from the GeneBank were submitted to the Multiple Sequence Alignment CLUSTALW program of the Kyoto University Bioinformatics Center. The divergence in amino acid sequences can be separated into two different groups. Various species of *Drosophila* AP-4 protein with high similarity in sequence were clustered together with other species of insects and grouped as Clade A. While other AP-4 proteins from *Saccoglossus*, a hemichordate, to mammals (at the bottom of the cluster) were clustered as Clade B.

Figure S2. *crp* mRNA expression

In situ hybridizations showing the *crp* RNA distribution in dissected tissues from third-instar larvae (A-D) and in whole-mount embryos (E-H). *crp* RNA is detected in most larval tissues. (A) High levels of *crp* are detected in the midgut endoderm and the imaginal histoblasts of the gut. (B) All of the imaginal discs express very high levels of *crp* RNA. (C) *crp* RNA is observed in the pericardial cells (indicated by the arrows) and, at lower levels, in the cardiac cells (arrowhead) of the heart. *crp* RNA is expressed at very low levels in the fat body, observed just below the heart. (D) The epidermal cells also express very low levels of *crp* RNA. (E) A stage 5 embryo. Note the ubiquitous expression of *crp* RNA. (F) A stage 8 embryo. *crp* RNA is broadly

distributed, with higher levels in invaginating tissues and the developing nervous system. (G) A stage 11 embryo. *crp* RNA is expressed at high levels in the developing gut and the nervous system. (H) A stage 15 embryo, high levels of *crp* RNA are restricted to the central nervous system.

Figure S3. Crp protein expression is ubiquitous.

In almost all of the tissues examined, except the epidermis, Cropped is primarily localized in the nuclei of many tissues. (A) The heart. Crp protein is detected in the nuclei of pericardial cells. (B) The wing imaginal disc. (C) The epidermis. Crp protein is detected at low levels. (D) A somatic muscle. (E) A tracheal terminal cell, indicated by an arrow head. (F) The dorsal trunk of the trachea (arrowhead). (G) Midgut endodermal cells.

Figure S4. Overexpression of actin and Ds-RED protein do not affect terminal branching

Specific expression of actin (A) and DsRed protein (B) in the terminal cells did not affect the branching patterns in DB and LG. Using *bs-Gal4*, actin and Ds-Red were specifically overexpressed in the terminal cells to demonstrate that the *Gal4/UAS* expression system did not cause any tracheal branching phenotype.

Figure S5. dMyc induces tortuous terminal branches

The terminal branching phenotypes in larvae overexpressing dMyc in the terminal cells by *bs-Gal4* and in larvae of *UAS-dm* L3 alone as control and. The photographs in the upper panels (A and B) show the terminal branches in the dorsal branches (DB), and those in the lower panel (C and D) were LG branches. The white arrow head in B points to the extra thick and tortuous terminal branch circled around the terminal nucleus/cell body and even the stalk of DB itself was not smooth as compared with that of *UAS-dm* larvae (A). The fine terminal branches (white arrow heads) of LG branches in *dm*-overexpressing larvae (D) were more tortuous than those of WT (C).

Figure S6. Alignment of AP-4 and Myc binding sites on the *cropped* genes of different *Drosophila* species

A schematic diagram shows the locations of Myc binding sites (red) and Crp/AP-4 binding sites (green) along the promoter and coding region of the *crp* genes of four species of *Drosophila*, and the alignment with those of *D. virilis*, and *D. pseudoobscura* were not drawn. The position of the transcription start site (TSS) is only known and marked in *Drosophila melanogaster*. The numbers that appear above the lines are the nucleotide positions of the sites, and the translation start site (ATG) is arbitrarily designated as +1. The protein coding region of the *crp* gene is represented by the shiny gray rectangles. The gray dotted lines show the corresponding AP-4 binding sites between two species. The only Myc binding site in the four species is present at -55 bp from the translation start site. The blue bar below the promoter of *Drosophila melanogaster* marks the DNA sequence of the *crp* gene used in the promoter activity assay.

Figure S1

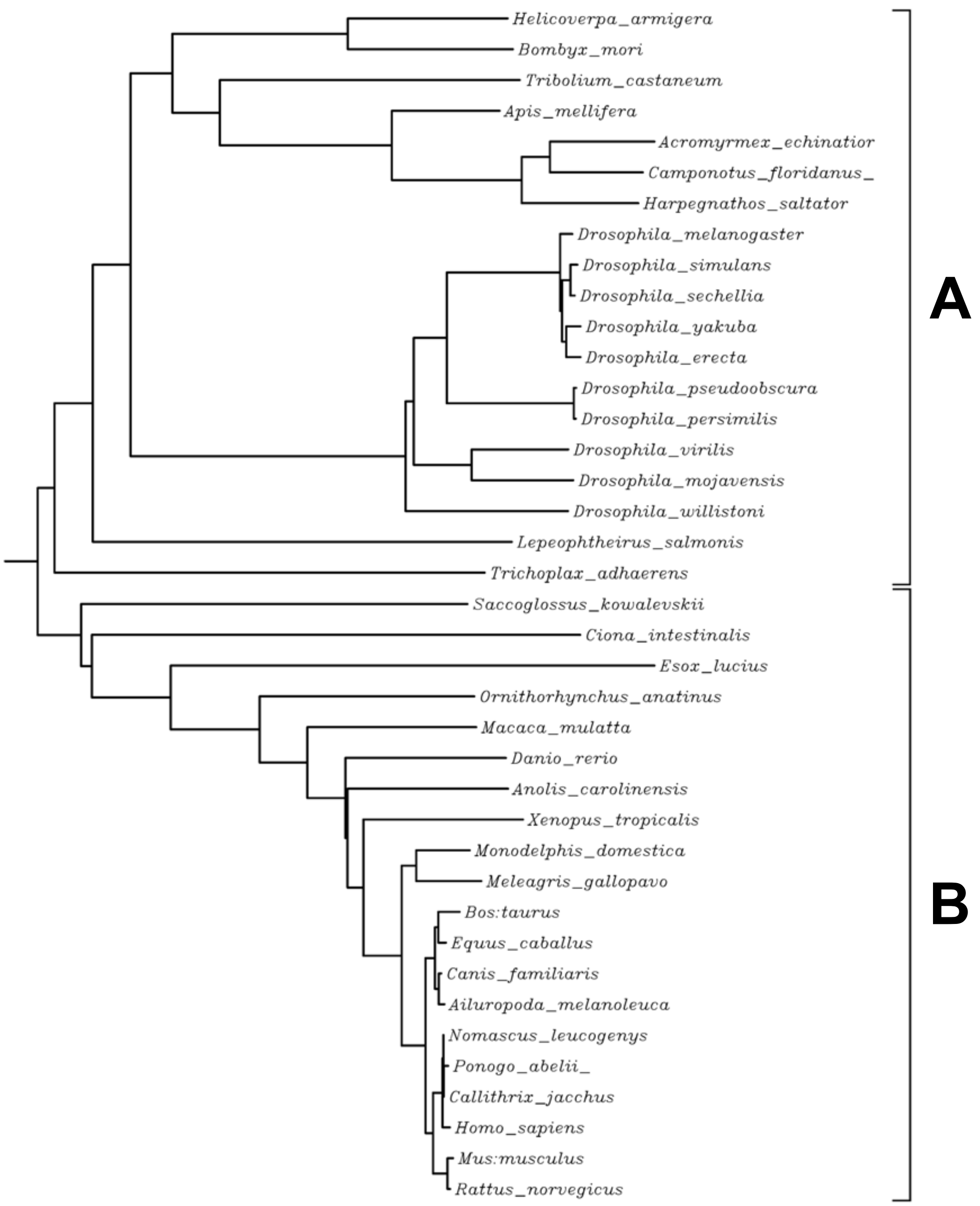

Helicoverpa_armigera
Bombyx_mori
Tribolium_castaneum
Apis_mellifera
Acromyrmex_echinatior
Camponotus_floridanus_
Harpegnathos_saltator
Drosophila_melanogaster
Drosophila_simulans
Drosophila_sechellia
Drosophila_yakuba
Drosophila_erecta
Drosophila_pseudoobscura
Drosophila_persimilis
Drosophila_virilis
Drosophila_mojavensis
Drosophila_willistoni
Lepeophtheirus_salmonis
Trichoplax_adhaerens
Saccoglossus_kowalevskii
Ciona_intestinalis
Esox_lucius
Ornithorhynchus_anatinus
Macaca_mulatta
Danio_rerio
Anolis_carolinensis
Xenopus_tropicalis
Monodelphis_domestica
Meleagris_gallopavo
Bos:taurus
Equus_caballus
Canis_familiaris
Ailuropoda_melanoleuca
Nomascus_leucogenys
Ponogo_abelii_
Callithrix_jacchus
Homo_sapiens
Mus:musculus
Rattus_norvegicus
A
B

Figure S2

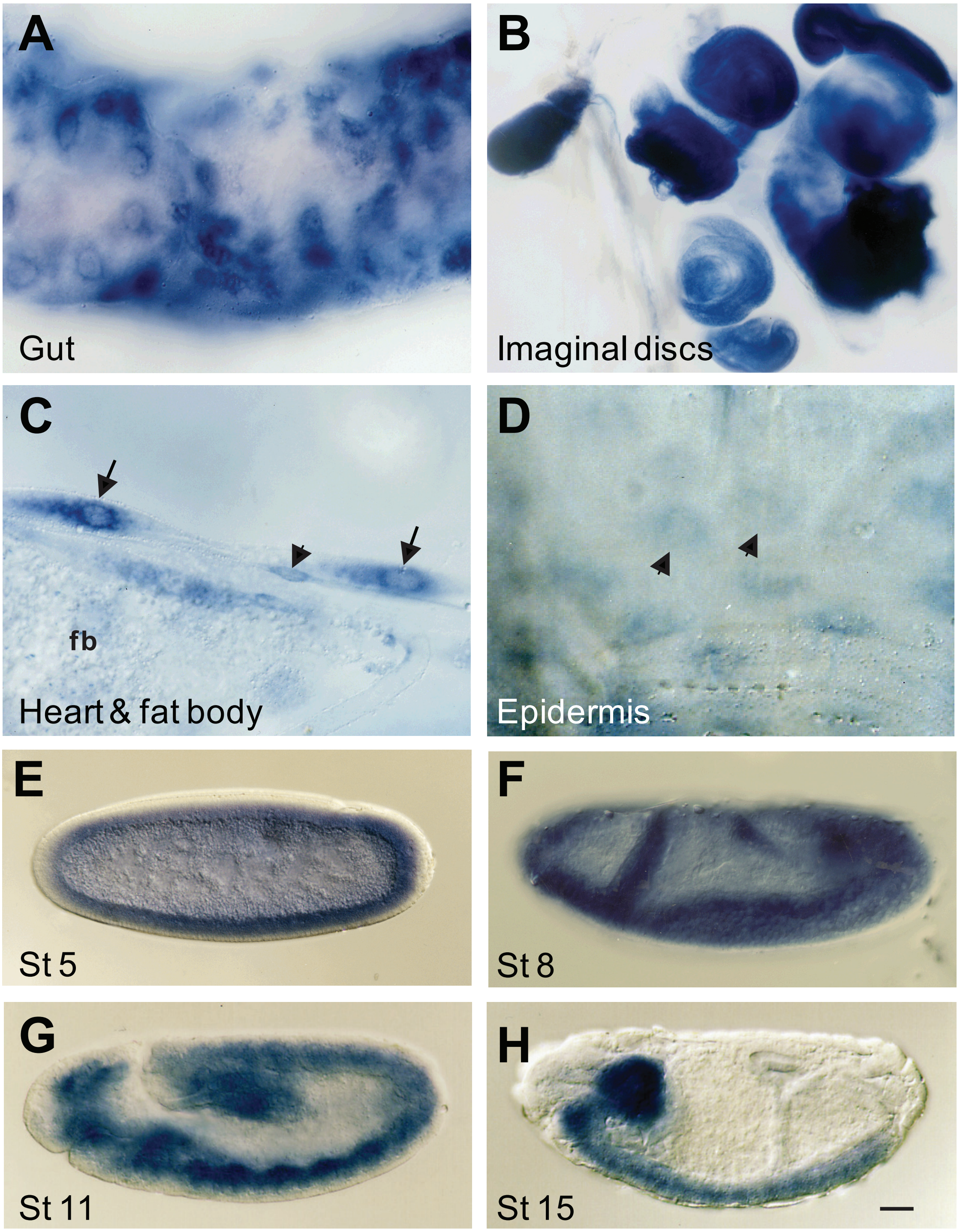
A
Gut
B
Imaginal discs
C
fb
Heart & fat body
D
Epidermis
E
St 5
F
St 8
G
St 11
H
St 15

Figure S3

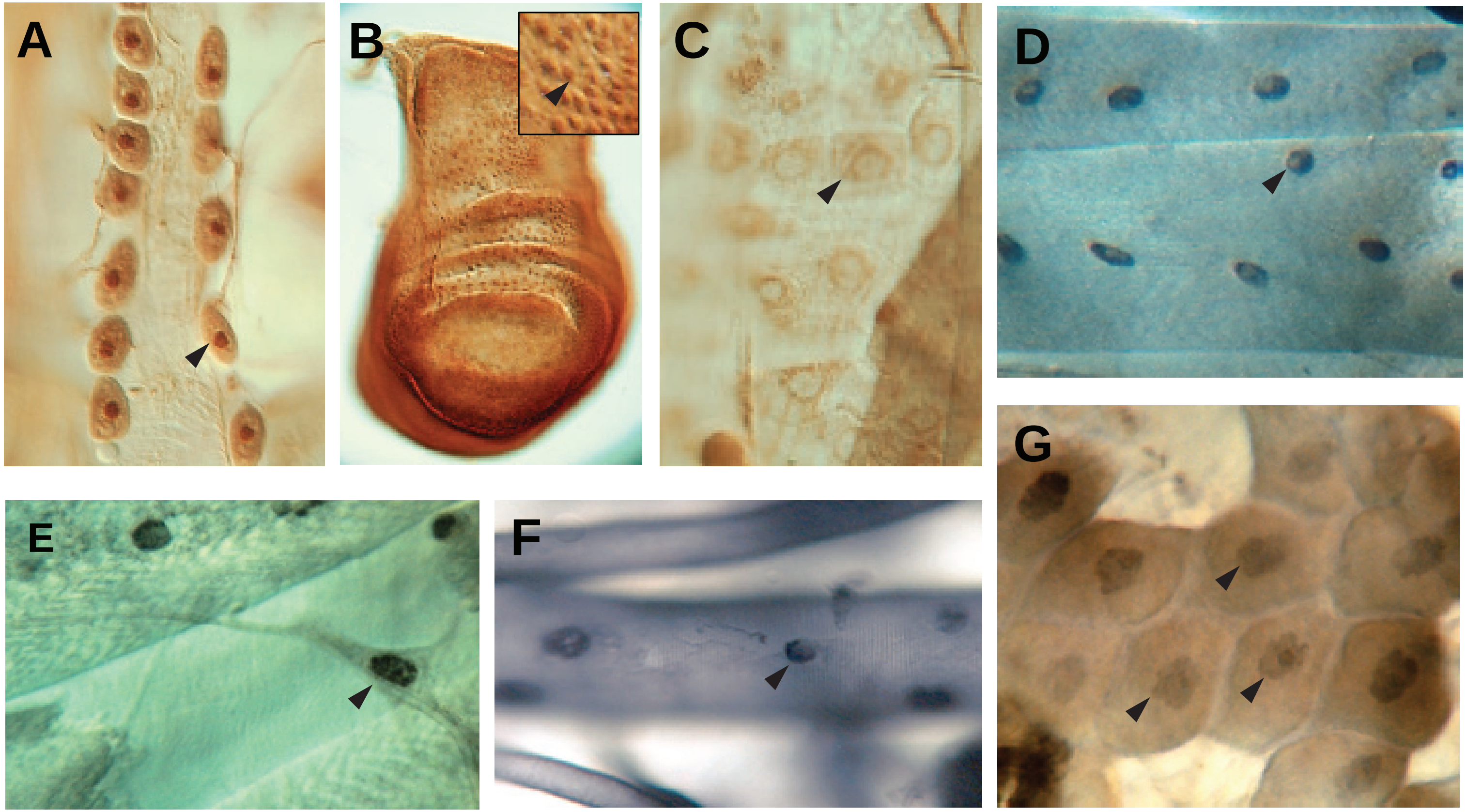

Figure S4

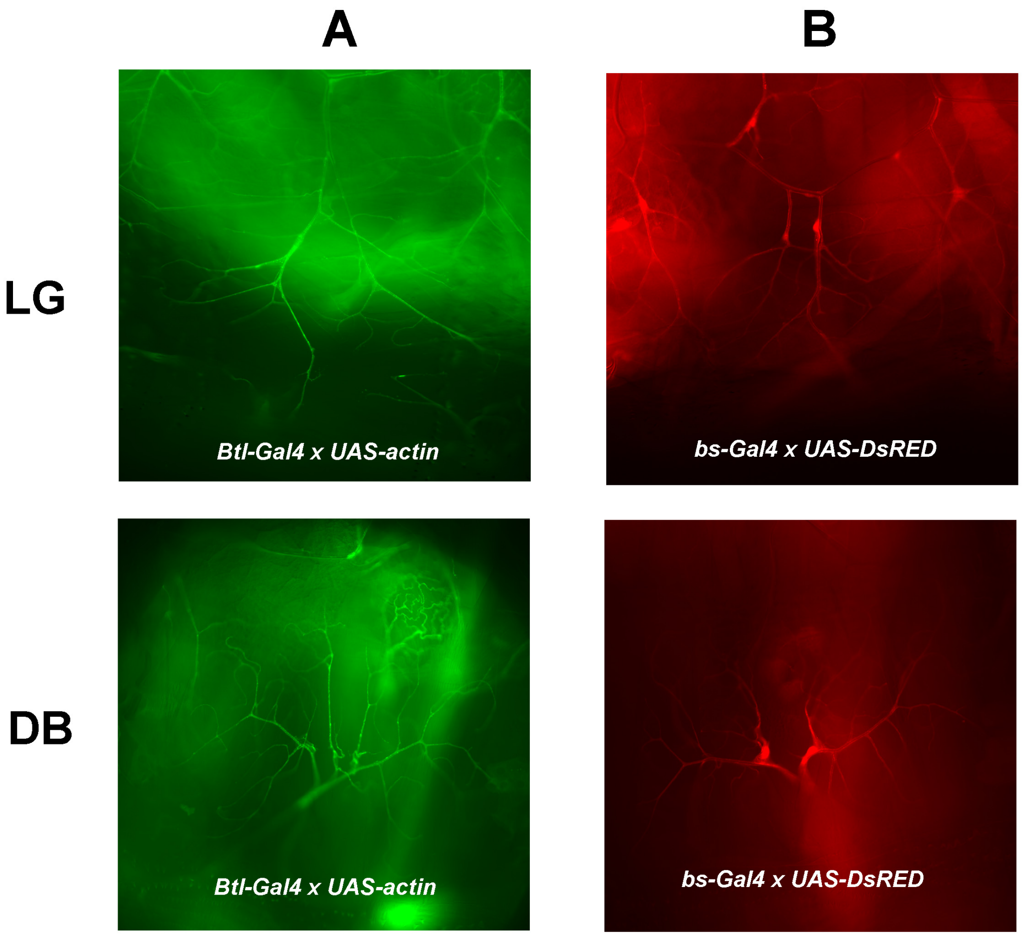
A
B
LG
DB
Btl-Gal4 x UAS-actin
bs-Gal4 x UAS-DsRED
Btl-Gal4 x UAS-actin
bs-Gal4 x UAS-DsRED

Figure S5

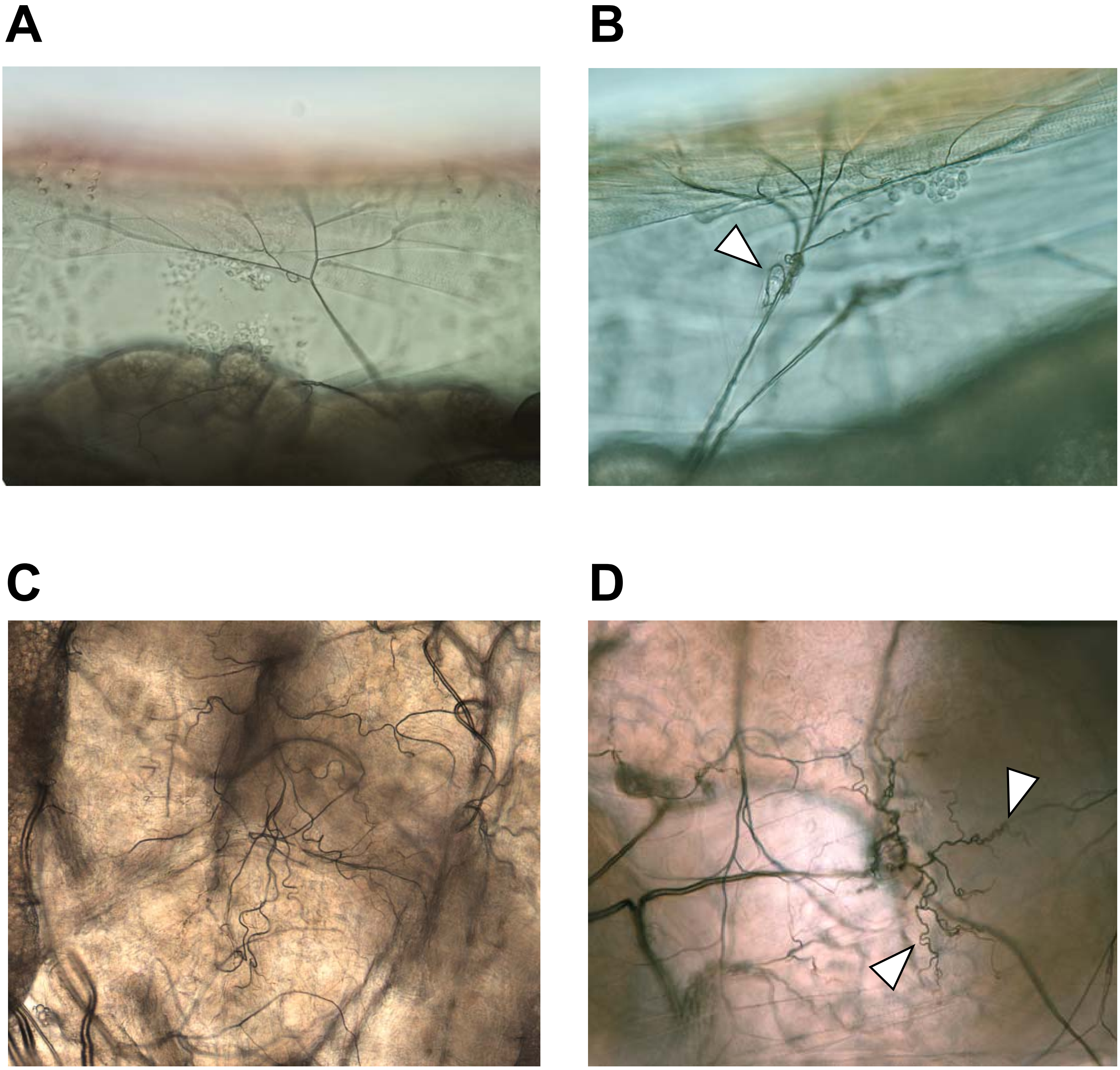

Figure S6

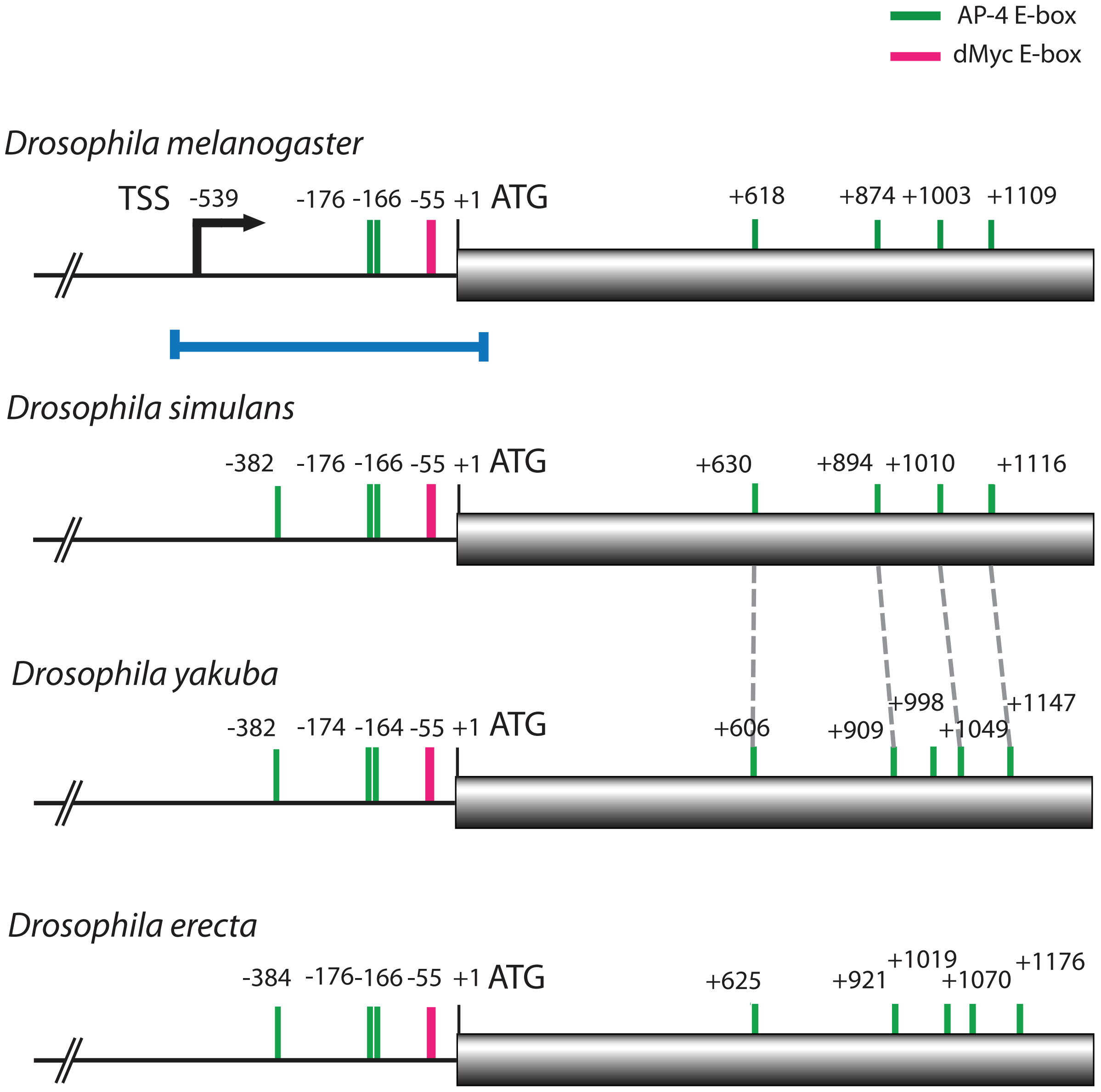

AP-4 E-box
dMyc E-box
Drosophila melanogaster
TSS
-539
-176
-166
-55
+1
ATG
+618
+874
+1003
+1109
Drosophila simulans
-382
-176
-166
-55
+1
ATG
+630
+894
+1010
+1116
Drosophila yakuba
-382
-174
-164
-55
+1
ATG
+606
+909
+998
+1049
+1147
Drosophila erecta
-384
-176
-166
-55
+1
ATG
+625
+921
+1019
+1070
+1176
